# Case Report: Cutaneous niche-restricted malignant phenotypes in PTCL-NOS revealed by single-cell sequencing

**DOI:** 10.3389/fonc.2026.1827539

**Published:** 2026-05-26

**Authors:** Shucheng Zhang, Zhuqing Li, Yang Lin, Xiaoyue Sun, Meng Qiao, Xiaoqing Si

**Affiliations:** 1 Department of Dermatology, The First Affiliated Hospital of Shandong First Medical University, Jinan, China; 2 China Foundation for Youth Entrepreneurship and Employment-Incaier Public Welfare Fund, Beijing, China; 3 Shandong Key Laboratory of Digital Diagnosis and Treatment of Thoracic Oncology, Shandong Engineering Research Center of Intelligent Surgery, Shandong Data Open Innovative Application Laboratory, The First Affiliated Hospital of Shandong First Medical University& Shandong Provincial Qianfoshan Hospital, Jinan, China; 4 School of Traditional Chinese Medicine, Shandong University of Traditional Chinese Medicine, Jinan, China

**Keywords:** cutaneous lymphoma, metabolic reprogramming, NF-κB signaling, PTCL-NOS, single-cell RNA sequencing, treatment resistance, tumor microenvironment

## Abstract

Peripheral T-cell lymphoma, not otherwise specified (PTCL-NOS) presenting with disseminated cutaneous involvement represents an aggressive disease subset with poor response to conventional chemotherapy and dismal prognosis. The tissue-specific molecular mechanisms driving cutaneous aggression remain poorly characterized at single-cell resolution. Here we report a 67-year-old male PTCL-NOS patient with hypertension and coronary artery disease history, presenting with generalized cutaneous nodules and rapid progression despite multi-line chemotherapy. Single-cell RNA sequencing (scRNA-seq) performed on paired skin lesions and peripheral blood at diagnosis revealed striking microenvironmental dichotomy: skin-resident malignant T cells exhibited hyperproliferative phenotypes (high CDC20B, HIST1H3B, MKI67+), activation of NF-κB and IL-17 signaling, and extensive crosstalk with endothelial cells; conversely, blood-derived lymphoma cells displayed immune evasion signatures and metabolic stress markers. Copy number variation analysis confirmed clonal expansion across both compartments with distinct tissue-specific transcriptional programs. Despite CHOP, CHOEP, DA-EPOCH, and AC-CHOP (azacitidine plus chidamide) regimens, the patient experienced primary refractory disease and died 6 months from diagnosis following COVID-19 superinfection. To our knowledge, this is the first case reporting single-cell transcriptomic comparison of skin versus blood compartments in PTCL-NOS, revealing how cutaneous microenvironment sculpts aggressive malignant phenotypes and providing potential targets for compartment-specific therapy.

## Introduction

Peripheral T-cell lymphomas (PTCL) comprise a heterogeneous group of aggressive non-Hodgkin lymphomas arising from post-thymic T lymphocytes, accounting for approximately 15–20% of lymphoid neoplasms (1, 2). Among these, PTCL-NOS represents the most common subtype; however, the lack of specific classification criteria, coupled with nonspecific clinical features and an aggressive course, renders diagnosis particularly challenging. In contrast, cutaneous T-cell lymphomas (CTCLs) are rare, constituting only 1–2% of non-Hodgkin lymphomas (3, 4). Primary cutaneous PTCL-NOS (pc-PTCL-NOS), an exceedingly rare variant comprising merely 2% of CTCLs, is characterized by a particularly poor prognosis, as evidenced by a 5-year disease-specific survival of only 15% (5). While pc-PTCL-NOS is defined by skin-limited disease without extracutaneous involvement at diagnosis, our patient presented with concurrent lymphadenopathy, bone marrow infiltration, and widespread visceral involvement, consistent with systemic PTCL-NOS with secondary cutaneous spread.

The tumor microenvironment (TME) plays a critical role in PTCL pathogenesis (6, 7). Oncogenic programs collaborate with the microenvironment to promote lymphomagenesis through immune evasion mechanisms, including recruitment of tumor-associated macrophages (TAMs) and regulatory T cells (6, 8, 9). However, the cellular heterogeneity and intercellular communication networks within cutaneous PTCL lesions remain largely unexplored at single-cell resolution. Single-cell RNA sequencing (scRNA-seq) offers an unprecedented opportunity to dissect the transcriptomic landscape of malignant T cells and their interactions with surrounding stromal and immune compartments (10).

Here we report a comprehensive single-cell transcriptomic analysis of a rare case of PTCL-NOS presenting with generalized cutaneous nodules and primary refractory disease. By comparing paired skin and blood samples obtained at diagnosis, we reveal a striking bifurcation in the tumor microenvironment, with distinct transcriptional programs, cellular interactions, and metabolic states shaping malignant T-cell behavior in these two compartments.

## Case presentation

In our study, we reported a 67-year-old male treated at The First Affiliated Hospital of Shandong First Medical University, Jinan, Shandong Province, China, who experienced a two-month history of progressive, painless, non-pruritic erythematous papules on the abdomen that rapidly disseminated throughout the body, accompanied by peripheral edema. The patient had a 30-year history of hypertension (grade 2, very high risk) and a history of myocardial infarction with coronary stent implantation 11 years prior, currently on aspirin and isosorbide mononitrate. He had completed three doses of an inactivated COVID-19 vaccine, with the third dose administered in February 2022.

**Figure 1:**
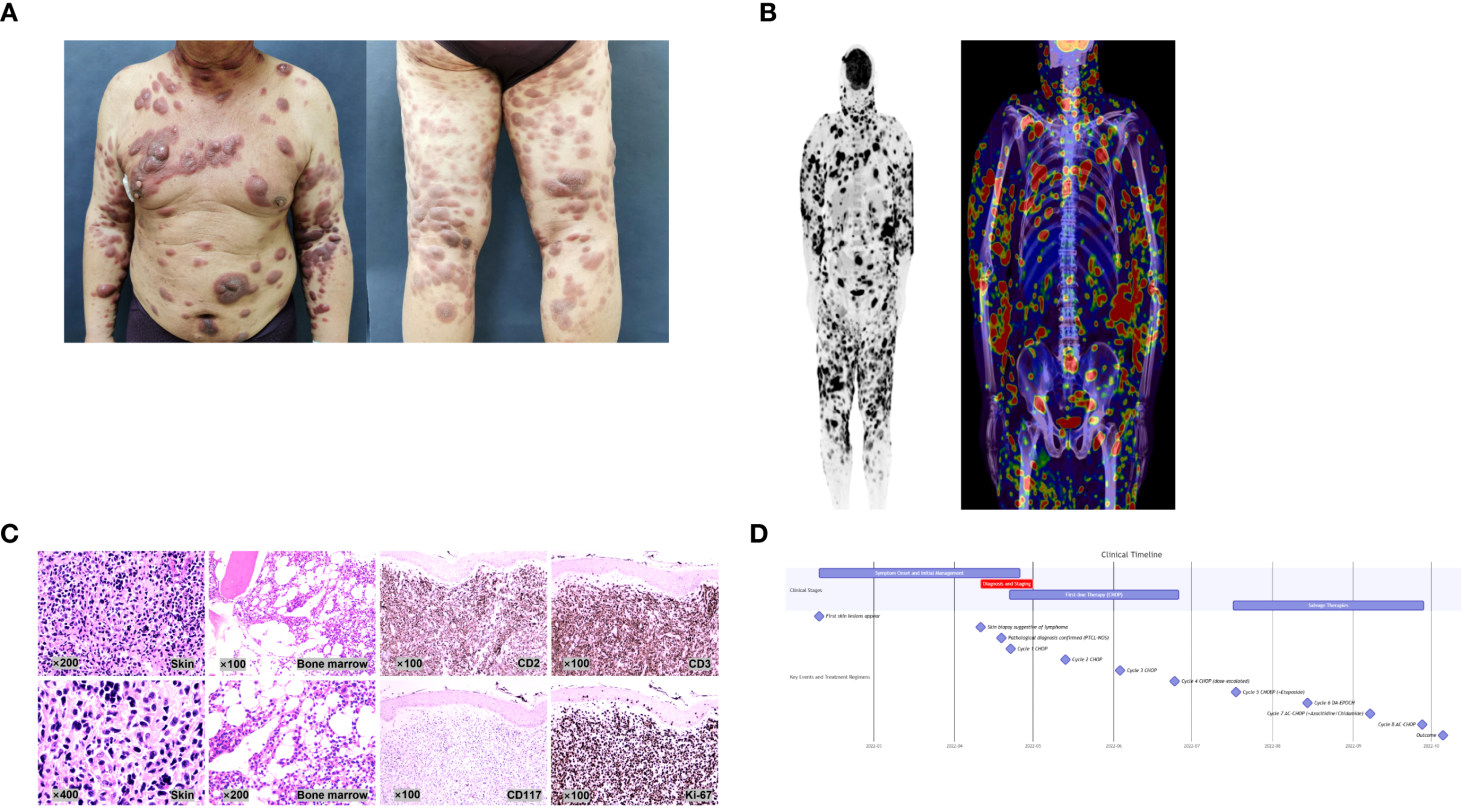
Clinical and pathological features of PTCL-NOS with cutaneous involvement. **(A)** Clinical photograph showing disseminated erythematous nodules and plaques. **(B)** PET-CT maximum intensity projection revealing widespread hypermetabolic lesions in skin, lymph nodes, and viscera. **(C)** The first column shows the H&E staining of skin biopsy sections at ×200 (top) and ×400 (bottom) magnification. The staining identifies dense dermal infiltration by medium-to-large atypical T cells with irregular nuclear contours. The second column shows the H&E staining of bone marrow biopsy sections at ×100 (top) and ×200 (bottom) magnification, revealing infiltration by atypical lymphoid cells. The third and fourth columns were immunohistochemistry of skin lesions, suggesting CD2+, CD3+, CD117− tumor cells, with Ki-67 proliferation index approximately 90%. **(D)** Clinical timeline illustrating disease course, treatment regimens, and outcomes.

Diagnostic trajectory: The patient was initially misdiagnosed at external institutions as having “allergic dermatitis” and subsequently “pityriasis rosea” (February 2022), receiving corticosteroids without improvement. Skin biopsy performed at Heze Municipal Hospital on April 11, 2022 revealed atypical lymphoid infiltration suspicious for lymphoma. The patient was admitted to our institution on April 14, 2022. Physical examination revealed diffuse infiltrative dark-red to purple nodules ranging from 1–5 cm in diameter, with firm consistency and peripheral edema (**Figure 1A**). Definitive diagnosis and staging: Laboratory investigations revealed lymphopenia [0.48×10^9^/L; reference range (RR): typically 1.1–3.2×10^9^/L] and relative monocytosis (1.36×10^9^/L; 15.5% of leukocytes), alongside neutrophilia (6.73×10^9^/L; 76.9%). Hypoproteinemia was evident with decreased total protein (59.30 g/L), albumin (39.80 g/L), and globulin (19.50 g/L) levels. Tumor markers showed elevation of CA-125 (56.9 U/mL), CA72-4 (7.55 U/mL), and neuron-specific enolase (28.10 ng/mL). Hypercoagulability was indicated by markedly elevated D-dimer (8.11 mg/L) and fibrin degradation products (21.03 mg/L). Immunological assessment revealed low serum IgM (<0.178 g/L) and positive antinuclear antibodies (ANA) at a titer of 1:100 with nucleolar pattern. PET-CT demonstrated widespread hypermetabolic skin lesions (SUVmax 2.5–40.9), lymphadenopathy (cervical, axillary, inguinal; SUVmax 1.3–3.9), consistent with Ann Arbor Stage IV disease (**Figure 1B**). Skin biopsy showed dense dermal infiltration by medium-to-large atypical lymphoid cells with irregular nuclear contours. Immunohistochemistry demonstrated: CD2+, CD3+, CD4+, CD5+, CD7−, CD8+ (partial), CD20−, CD30−, CD56−, CD117−, GrB−, ALK−, TIA-1+, Ki-67≈90%; EBER *in situ* hybridization was negative (**Figure 1C**). Bone marrow biopsy revealed 9% undifferentiated cells with atypical morphology. TCR gene rearrangement showed polyclonality.

Treatment course and refractory disease: The patient was transferred to the Department of Hematology on April 20, 2022, and initiated chemotherapy:Following diagnosis, the patient received CHOP chemotherapy (cyclophosphamide 1.2g, doxorubicin 40mg, vindesine 4mg, prednisone 100mg days 1–5). After cycles 1–2, partial response was observed with flattening of skin lesions. However, by the fifth admission (July 15, 2022), cutaneous nodules showed progressive increase in size and number, with lesions becoming violaceous to black-purple in color, indicating primary refractory disease. Salvage regimens were sequentially administered. CHOEP (addition of etoposide 0.2 g days 1–2) was given on July 17, 2022, followed by DA-EPOCH (dose-adjusted continuous infusion protocol) on August 12, 2022. Given continued progression, AC-CHOP combining azacitidine (200 mg days 1–2, 100 mg day 3) and chidamide (HDAC inhibitor, 20 mg twice weekly) with CHOP backbone was administered on September 5 and 26, 2022. Despite these intensive regimens, the patient developed rapidly progressive disease with prominent necrotic-appearing nodules on the forehead and pharyngeal area (7 cm diameter), severe pancytopenia (WBC 2.64×10^9^/L, hemoglobin 115 g/L), and febrile neutropenia. Unfortunately, the patient developed severe COVID-19 pneumonia during profound chemotherapy-induced immunosuppression. Given severe pancytopenia and febrile neutropenia, he received supportive care only, including supplemental oxygen, antipyretics, and fluid management. His respiratory status rapidly deteriorated, and he died on October 1, 2022, approximately 6 months from diagnosis (**Figure 1D**).

### Single-cell transcriptomic analysis

**Figure 2:**
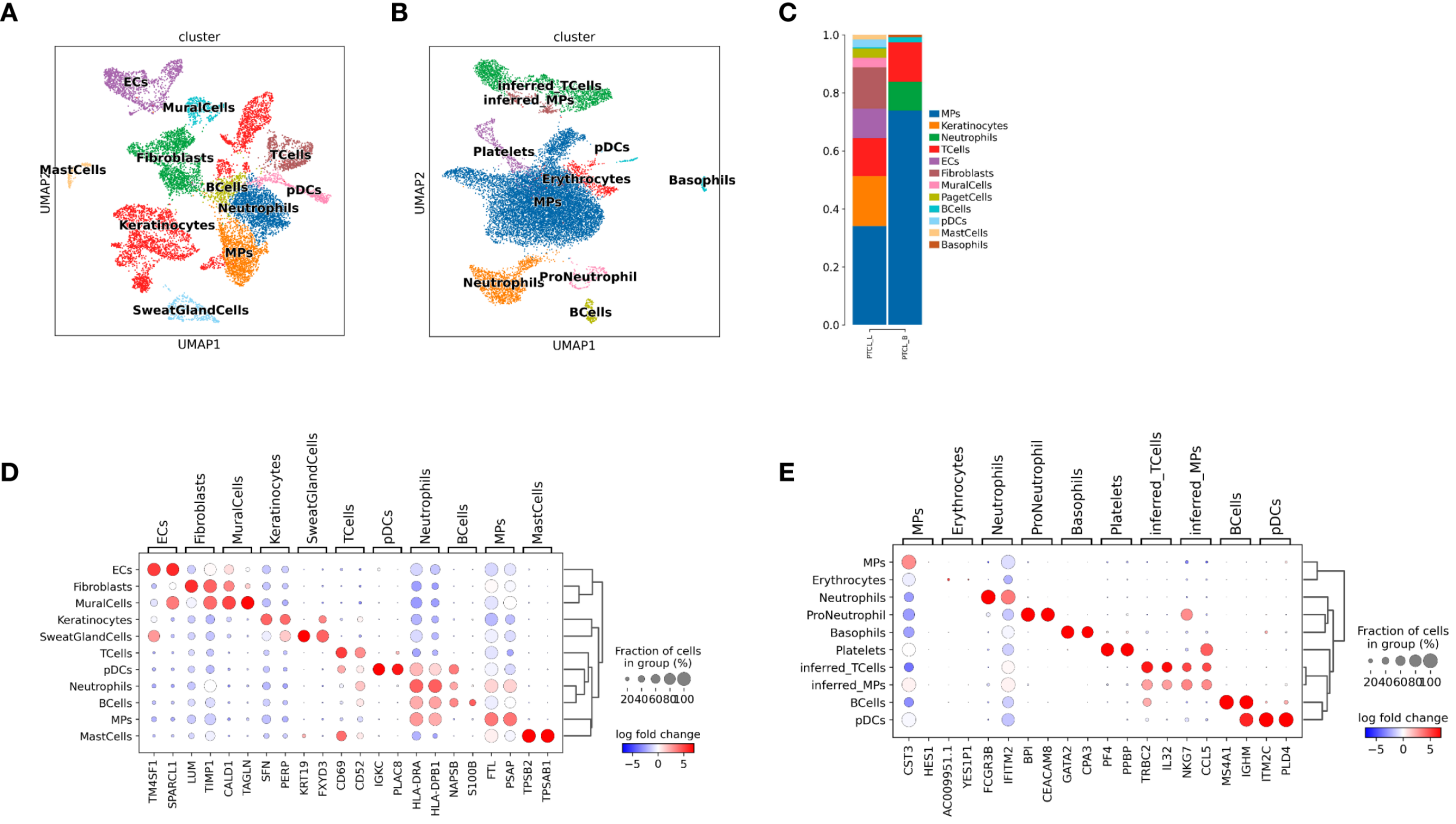
Single-cell transcriptomic landscape of skin and blood compartments. **(A)** Skin (PTCL_L): UMAP visualization of 12, 262 cells from skin lesion colored by cell type, showing distinct clusters of T cells (TCs), keratinocytes (KCs), endothelial cells (ECs), fibroblasts (FBs), mural cells (MCs), macrophages (MPs), neutrophils, B cells, plasma dendritic cells (pDCs), mast cells, and sweat gland cells. **(B)** Blood (PTCL_B): UMAP visualization of 16, 285 cells from peripheral blood, displaying inferred T cells, MPs, erythrocytes, neutrophils, platelets, B cells, pDCs, basophils, and pro-neutrophils. **(C)** Stacked bar plot comparing cellular composition between Skin (PTCL_L) and Blood (PTCL_B) samples, revealing compartment-specific enrichment of keratinocytes and fibroblasts in skin versus erythrocytes and neutrophils in blood. **(D)** Marker gene expression dot plot validating cell type identities in skin. **(E)** Marker gene expression dot plot for blood cell populations.

To dissect the cellular heterogeneity and microenvironmental dichotomy underlying the aggressive cutaneous phenotype, we performed scRNA-seq on paired skin lesions (A2) and peripheral blood (A3) obtained at diagnosis (April 14–20, 2022) prior to chemotherapy initiation using the 10× Genomics Chromium platform. Due to ethical constraints and the patient’s near-total body surface involvement by dense infiltrative nodules, only lesional skin tissue was obtained; normal skin tissue was not biopsied. After rigorous quality control, we analyzed 12, 262 cells from skin and 16, 285 cells from blood. Unsupervised clustering revealed 11 distinct cell populations in skin and 10 in blood (**Figures 2A, B**). The skin microenvironment was dominated by keratinocytes (26.9%), followed by fibroblasts (14.2%), neutrophils (14.9%), and macrophages (MPs, 12.1%), alongside substantial populations of endothelial cells (ECs, 10.2%), T cells (6.2%), and B cells (3.8%) (**Figure 2C**). In stark contrast, peripheral blood exhibited overwhelming macrophage predominance (MPs, 68.1%), with inferred malignant T cells representing a notable subset (11.7%), alongside neutrophils (8.7%), erythrocytes (3.0%), and platelets (2.9%) (**Figures 2B, C**). Cell type identities were validated by established lineage-specific markers (**Figures 2D, E**).

**Figure 3:**
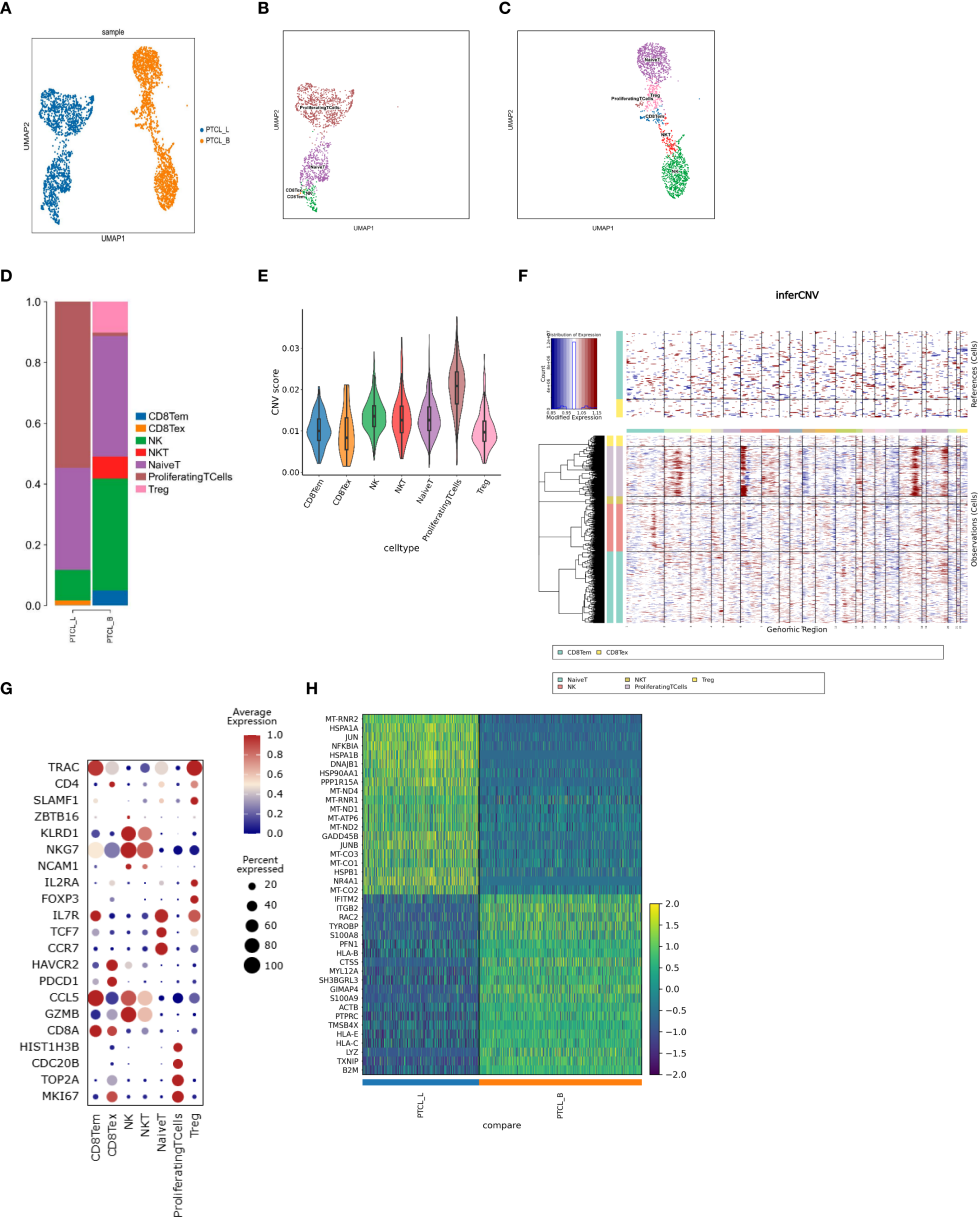
Characterization of malignant T-cell clones and tissue-specific transcriptional programs. **(A)** Integrated UMAP visualization of T/NK cells from both compartments, colored by tissue origin (Skin/PTCL_L in blue; Blood/PTCL_B in orange), demonstrating distinct spatial segregation of skin- versus blood-resident lymphocytes. **(B)** Skin-derived T cells: UMAP revealing six transcriptional subpopulations: proliferating T cells, naïve T cells, CD8+ effector memory T cells (CD8+ Tem), exhausted CD8+ T cells (CD8+ Tex), NK cells, and NKT cells. **(C)** Blood-derived T cells: UMAP showing distribution of proliferating T cells, naïve T cells, Tregs, CD8+ Tem, NKT cells, and NK cells. **(D)** Stacked bar plot quantifying the relative proportions of T-cell subpopulations between Skin (PTCL_L) and Blood (PTCL_B). **(E)** Violin plot of CNV scores across T-cell subtypes (analysis performed on integrated skin and blood T/NK cells), with proliferating T cells exhibiting significantly higher scores, confirming their malignant nature. **(F)** InferCNV heatmap displaying large-scale chromosomal copy number variations across T-cell subpopulations (integrated skin and blood samples), using CD8+Tem/CD8+Tex as reference, with hierarchical clustering revealing distinct CNV signatures. **(G)** Dot plot showing average expression (color scale) and percent of expressing cells (dot size) for selected marker genes across T-cell subpopulations (integrated skin and blood). Proliferating T cells co-express MKI67, TOP2A, and CDC20B, validating the high Ki-67 proliferation index observed in immunohistochemistry (**Figure 1C**). CD4 expression in proliferating T cells confirms the helper T-cell phenotype of the malignant clone, consistent with immunohistochemical findings. **(H)** Heatmap of differentially expressed genes between Skin (PTCL_L) and Blood (PTCL_B) T cells, showing upregulation of heat shock proteins (HSPA1A/B), unfolded protein response genes (PPP1R15A), and mitochondrial genes (MT-ND4, MT-CO1) in skin, versus enrichment of MHC class I molecules (HLA-B/C/E) and S100 family proteins in blood.

Focusing on the lymphoid compartment to distinguish malignant from reactive lymphocytes, we subsetted T/natural killer (NK) cells and performed integrative analysis. The UMAP projection revealed clear compartmental segregation, with skin-derived T cells forming distinct clusters separate from blood-derived counterparts (**Figures 3A–C**). Subclustering identified seven transcriptionally distinct subpopulations: proliferating T cells, effector memory CD8+ T cells (CD8+ Tem), exhausted CD8+ T cells (CD8+ Tex), naïve T cells, regulatory T cells (Tregs), NK cells, and NKT cells (**Figures 3D, G**). Copy number variation (CNV) analysis using CD8+Tem/CD8+Tex as reference confirmed the malignant nature of specific clusters, with proliferating T cells exhibiting the highest CNV scores (mean 0.024), significantly exceeding those of naïve T cells (0.012) and Tregs (0.011) (**Figure 3E**). The inferCNV heatmap revealed extensive chromosomal gains and losses across malignant populations, with particularly prominent amplifications in chromosomes 1, 6, and 18 (**Figure 3F**), consistent with known karyotypic abnormalities in PTCL-NOS. Strikingly, the tissue distribution of malignant subclones was highly skewed: proliferating T cells constituted 54.7% (877/1, 604) of skin-resident T cells but merely 1.1% (20/1, 870) in blood, whereas naïve T cells showed relatively balanced distribution with modest peripheral blood predominance (39.7% vs. 33.6% in skin) (**Figure 3D**). Notably, CD8+ Tex cells were exclusively detected in skin lesions (25 cells), while Tregs and NKT cells were blood-restricted (191 and 135 cells, respectively), suggesting distinct microenvironmental selection pressures driving tissue-specific phenotypic adaptation and immune cell trafficking patterns.

**Figure 4:**
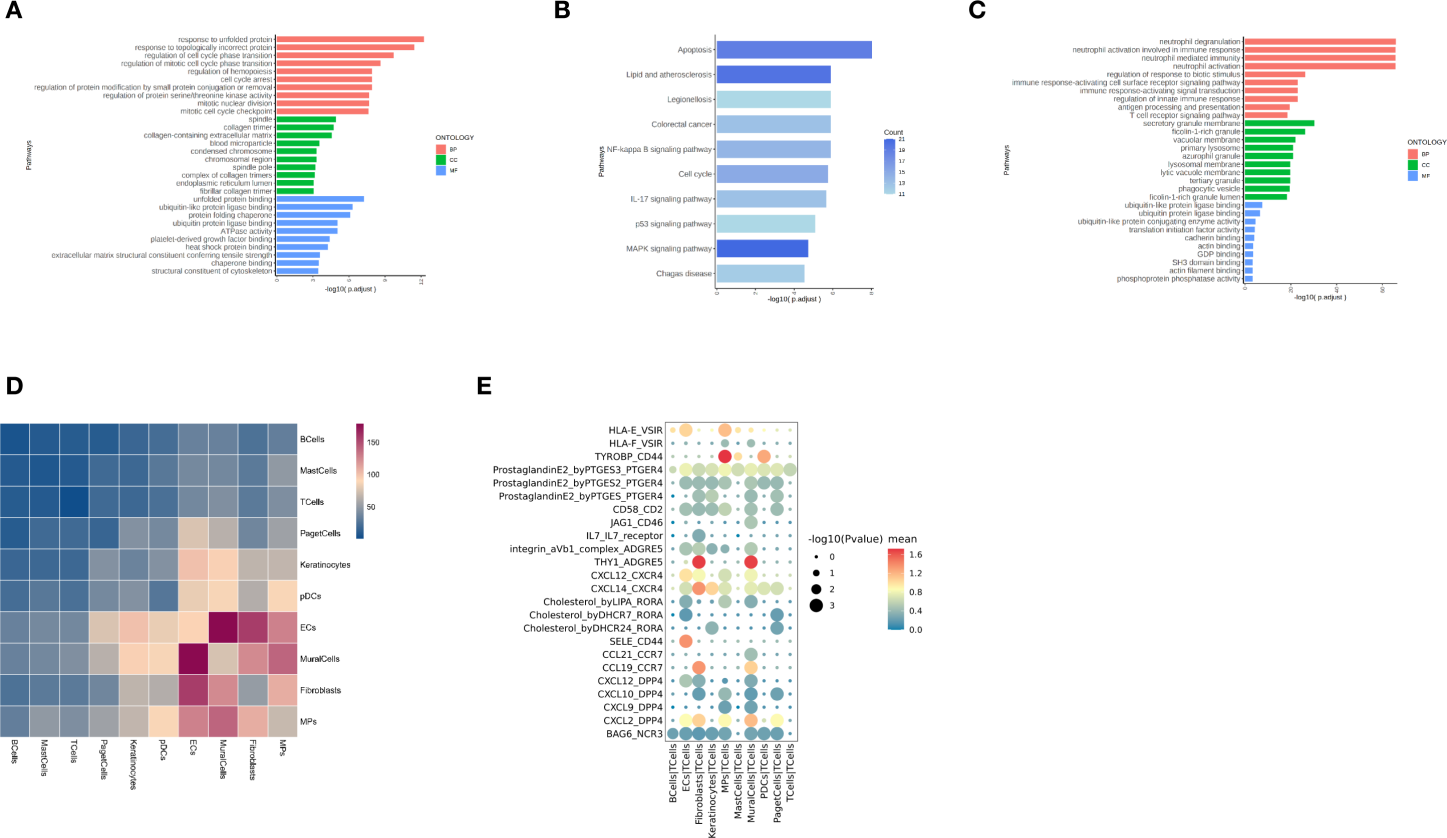
Pathway enrichment and cellular communication networks. **(A)** Skin (PTCL_L): GO enrichment analysis of upregulated genes in skin T cells, highlighting cell cycle regulation, unfolded protein response, and mitotic processes. **(B)** Skin (PTCL_L):KEGG pathway analysis showing enrichment of apoptosis, NF-κB signaling, IL-17 signaling, and cell cycle pathways. **(C)** Blood (PTCL_B): GO analysis of downregulated genes in blood T cells, showing suppression of neutrophil-mediated immunity and antigen presentation. **(D)** Skin (PTCL_L): Cell-cell communication heatmap showing interaction strength between T cells and other skin cell types, with strongest crosstalk observed with ECs, mural cells, and fibroblasts. **(E)** Skin (PTCL_L): Bubble plot showing significant ligand-receptor pairs between T cells (sender/receiver) and other cell types. Dot size represents -log10(P value) and color intensity indicates mean expression level. Key interactions include chemokine signaling (CXCL12/CXCL14-CXCR4, CCL19/CCL21-CCR7) and integrin-mediated adhesion (integrin αVβ1 complex-ADGRE5) between T cells and endothelial cells (ECs), fibroblasts, and mural cells.

Transcriptional profiling further revealed marked differences between compartments, with differential expression analysis identifying differentially upregulated genes in skin-resident malignant cells, including heat shock proteins (HSPA1A, HSPA1B, HSP90AA1), unfolded protein response genes (PPP1R15A, DNAJB1), and mitochondrial oxidative phosphorylation components (MT-ND4, MT-CO1, MT-ATP6), indicative of metabolic stress and high bioenergetic demands (**Figure 3H**). Conversely, blood-derived T cells upregulated major histocompatibility complex (MHC) class I molecules (HLA-B, HLA-C, HLA-E) and S100 family proteins (S100A8, S100A9), suggestive of immune surveillance evasion mechanisms. Gene Ontology (GO) enrichment analysis of skin-upregulated genes revealed significant enrichment in cell cycle regulation, response to unfolded protein, and protein kinase activity (**Figure 4A**), while Kyoto Encyclopedia of Genes and Genomes (KEGG) pathway analysis implicated apoptosis, NF-κB signaling, IL-17 signaling, and cell cycle control (**Figure 4B**), pathways consistently associated with chemotherapy resistance and aggressive lymphoma biology. In contrast, blood T cells exhibited downregulation of neutrophil-mediated immunity, antigen processing and presentation, and T cell receptor signaling pathways (**Figure 4C**), consistent with a quiescent, immune-evasive phenotype adapted for systemic circulation.

Beyond these cell-intrinsic transcriptional programs, cell-cell communication analysis using CellChat revealed extensive stromal networks within the skin microenvironment, with endothelial cells serving as the central hub interacting with mural cells and fibroblasts (**Figure 4D**). Detailed ligand-receptor interaction analysis further demonstrated that malignant T cells engaged in significant molecular crosstalk with ECs, fibroblasts, and mural cells (**Figure 4E**). Key communication axes included chemokine signaling (CXCL12/CXCL14-CXCR4, CCL19/CCL21-CCR7), integrin-mediated adhesion (integrin αVβ1 complex-ADGRE5), and immune checkpoint molecules (HLA-E-VSIR), which likely facilitate lymphoma cell trafficking, survival, and chemoresistance within the cutaneous niche.

## Discussion

PTCL-NOS with secondary cutaneous involvement represents an aggressive disease subset with limited therapeutic options and poor clinical outcomes. In this case, we utilized scRNA-seq to dissect the tissue-specific microenvironmental heterogeneity in disseminated systemic PTCL-NOS, revealing distinct transcriptional programs that correlate with the observed primary refractory phenotype. Our findings illuminate a striking dichotomy between skin and blood compartments in secondary cutaneous dissemination that may explain the primary refractory phenotype observed in this case. The cutaneous microenvironment promoted a highly proliferative malignant phenotype, with 54.7% of skin-resident T cells exhibiting proliferative signatures (CDC20B+, HIST1H3B+, MKI67+) compared to only 1.1% in peripheral blood. This proliferative advantage was accompanied by upregulation of heat shock proteins and unfolded protein response genes, suggesting that cutaneous infiltration imposes significant metabolic stress, selecting for stress-resistant clones capable of thriving in a nutrient-competitive, hypoxic niche (11, 12). This “cutaneous sanctuary” hypothesis aligns with the observed resistance to multiple chemotherapy regimens (CHOP, CHOEP, DA-EPOCH, and AC-CHOP), as high proliferative indices and metabolic plasticity are established mechanisms of chemoresistance in aggressive lymphomas (13–15). The activation of NF-κB and IL-17 signaling pathways in skin T cells aligns with previous reports implicating these pathways in T-cell lymphomagenesis and chemotherapy resistance (16–19). Our data extend these observations by demonstrating that NF-κB activation is compartment-specific, predominantly occurring in skin-resident rather than circulating lymphoma cells. This spatial restriction suggests that NF-κB inhibitors (e.g., bortezomib) may be particularly beneficial for controlling cutaneous lesions but may require combination with microenvironment-modulating agents to overcome stromal-mediated chemoresistance (20, 21).

In contrast to the proliferative skin phenotype, circulating lymphoma cells exhibited features of immune evasion, including upregulation of MHC class I molecules and S100 family proteins, potentially reflecting compensatory mechanisms to evade NK cell-mediated killing (22, 23). The downregulation of antigen presentation and T cell receptor signaling in blood-derived cells suggests a quiescent, “survival” phenotype optimized for immune surveillance evasion during systemic dissemination. This phenotypic plasticity—adopting a “growth” phenotype in skin versus a “survival” phenotype in blood—highlights the dynamic tumor evolution driven by tissue-specific selective pressures. The primary refractory nature of this case, evidenced by progression despite four cycles of CHOP and failure of intensive salvage regimens including HDAC inhibitors (chidamide) and hypomethylating agents (azacitidine), correlates with the extensive stromal crosstalk observed in skin lesions. The robust communication between malignant T cells and endothelial cells, mural cells, and fibroblasts through chemokine (CXCL12/CXCL14-CXCR4, CCL19/CCL21-CCR7) and integrin (integrin αVβ1 complex-ADGRE5) signaling creates a permissive microenvironment that promotes drug resistance through both cell-autonomous and non-cell-autonomous mechanisms. Notably, the patient ultimately succumbed to COVID-19 superinfection following severe immunosuppression, correlating with the observed systemic lymphocytopenia and exhausted T-cell phenotypes (PDCD1+, HAVCR2+) in peripheral blood. The scRNA-seq data revealed profound immune dysfunction, with blood-derived lymphoma cells exhibiting downregulated antigen presentation pathways and the microenvironment showing depleted effector immune populations. This highlights the dual therapeutic challenge in PTCL-NOS: achieving local control of aggressive cutaneous disease while preserving systemic immune competence to prevent infectious complications (24, 25).

In conclusion, this study represents the first single-cell transcriptomic comparison of skin versus blood compartments in PTCL-NOS, revealing how the cutaneous microenvironment sculpts aggressive, proliferative, and chemoresistant malignant phenotypes through NF-κB activation, metabolic reprogramming, and extensive stromal crosstalk. We acknowledge that the absence of paired normal skin tissue limits our ability to fully distinguish microenvironmental changes driven by malignant infiltration from baseline cutaneous stromal signatures; future studies incorporating normal skin controls from matched anatomical sites are warranted. These insights provide a mechanistic framework for developing compartment-specific therapeutic strategies, such as combining conventional chemotherapy with NF-κB inhibitors or metabolic modulators, to improve outcomes in this challenging lymphoma subtype.

## Data Availability

The original contributions presented in the study are included in the article/supplementary materials. Further inquiries can be directed to the corresponding author.
